# Delineating species in the speciation continuum: A proposal

**DOI:** 10.1111/eva.12748

**Published:** 2019-03-25

**Authors:** Nicolas Galtier

**Affiliations:** ^1^ UMR 5554 – Institut des Sciences de l'Evolution CNRS – University of Montpellier – IRD – EPHE Montpellier France

**Keywords:** gene flow, *Homo neanderthalensis*, speciation genomics, species barriers, species problem, systematics

## Abstract

Delineating species is a difficult and seemingly uninteresting issue that is still essential to address. Taxonomic methodology is heterogeneous according to the taxa and scientists involved due to the disparate data quality and quantity and disagreements over the species concept. This has negative impacts on basic and applied research. Genomic data substantially enhance our understanding of the speciation process but do not provide a ubiquitous solution to the species problem. The relevance of comparative approaches in speciation research has nevertheless recently been demonstrated. I suggest moving towards a more unified taxonomic classification through a reference‐based decision procedure.

## 1 INTRODUCTION

That living organisms should be pooled into species groups is a very intuitive idea, for at least two obvious reasons. First, the distribution of biological variation in nature is multimodal. A given organism typically resembles organisms within its own species much more than it does organisms from different species, and sharp boundaries can often be perceived. Secondly, only organisms that resemble each other can mate and generate offspring, which in turn resemble the parents. Identifying and naming species is a goal of anyone observing nature, and a prerequisite for any biological investigation—scientists need to know, and communicate about, the species they work on. Paradoxically, although the existence of species boundaries is quite intuitive, species delineation is typically a difficult task, even for specialists. Here, I discuss the reasons for this paradox in the light of recent genome‐wide data and analyses.

## 2 “CALL THEM SPECIES OR SUBSPECIES, NO BIG DEAL”

**Figure 1:**
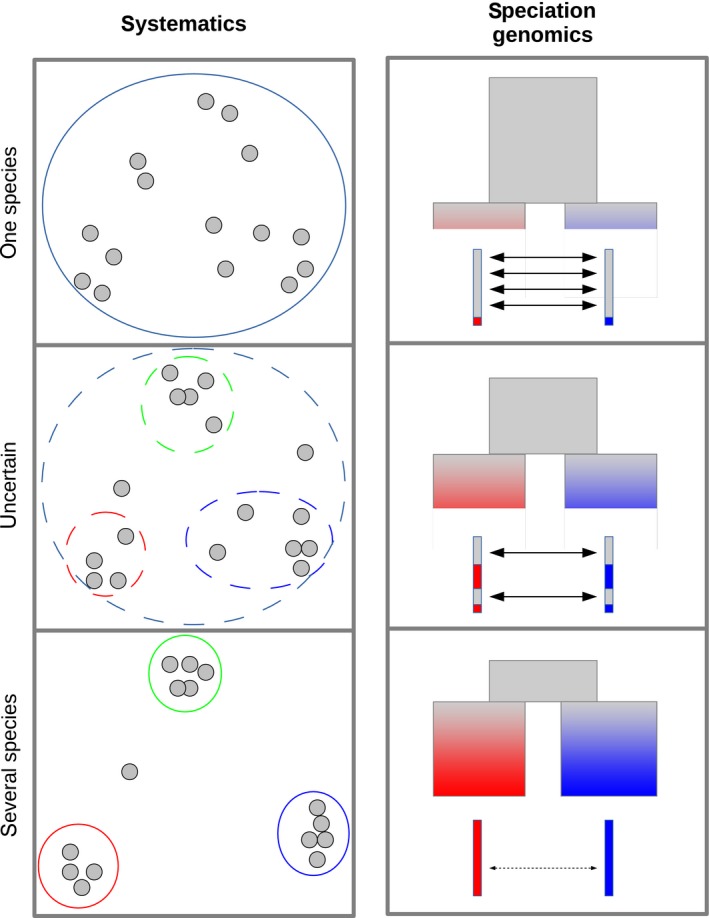
Two views on the continuum of speciation. Left: Species are defined as groups of organisms resembling each other according to an arbitrary set of variables. Right: Species are defined as entities sufficiently diverged such that gene flow (arrows) is very rare or inexistent. Top: unambiguous single‐species situation. Bottom: unambiguous multiple‐species situation. Intermediate: ambiguous situation. Ambiguous situations appear when groups can be identified but intermediate individuals are common (left) and when gene flow exists but is limited to a fraction of the genome (right)

Systematics involves studying relationships between living entities, and in the first place defining species. The main idea here is that, if nature is discontinuous, it should be possible to identify limits between clusters of organisms once the organisms have been described as thoroughly as possible, as illustrated in Figure 1a. This approach, however, has numerous limitations (Padial, Miralles, De la Riva, & Vences, 2010). It is necessary to determine which variables to consider, which is arbitrary and/or constrained by our ability to measure things. Morphology, for instance, is not equally powerful in all taxa. The risk of lumping sibling species is probably higher in small, colourless and/or morphologically simplified organisms, whereas there is a higher risk of over‐splitting large, colourful and complex organisms into separate taxonomic entities. Genetic data can help address this problem by providing ubiquitous, objective markers in any taxon of interest. A very large number of studies have attempted to reveal genetic clusters in specific taxa via multivariate analysis of randomly chosen, presumably neutral loci.

The use of genetic data, however, is far from solving the species delineation problem. One remaining obstacle, which also concerns morphological data, is that individuals do not always cluster in well‐defined entities (Figure 1a, middle panel). Intermediate individuals are frequently observed, and in many analyses, the cluster number and nature depend on arbitrary thresholds or parameters (Puechmaille, 2016). Yet another problem is that significant differences in allele frequencies may be found between entities that everybody recognizes as being distinct populations of a single species—genetic clusters and species are two different things (Mallet, 2007). The advent of genome‐wide data has emphasized this aspect: Statistical significance is readily reached when many loci are analysed (Jackson, Carstens, Morales, & O'Meara, 2017; Leaché, Zhu, Rannala, & Yang, 2018; Sukumaran & Knowles, 2017). The existence and nature of species boundaries in nature, however, are independent of the amount of data available to scientists. Genetic clusters are still of interest per se, irrespective of species limits. They can be informative on the strength of genetic drift, or on past or present gene flow reduction events, sometimes associated with local adaptation. Indeed, many population geneticists are happy with identifying and analysing genetic clusters, whereas whether or not they should be called species, subspecies or populations is often considered to be an uninteresting secondary issue.

## 3 @WTF_R_SPECIES

Another field of research tightly connected to the species problem is speciation genetics, which is geared towards understanding the process of new species formation, i.e., how a single gene pool evolves into two (or more) distinct entities (Figure 1b). Reproductive isolation, that is, the ability of individuals from different entities to exchange genes (Mayr, 1942), is a key concept in the speciation genetics literature. If speciation means evolving from free gene flow to no gene flow, then the current amount of gene flow between two entities could be taken as a measure of the speciation stage (Hey & Pinho, 2012). Species delineation methods based on the multispecies coalescent model, for instance, identify species as entities between which genetic exchanges have been negligible compared to drift (Yang & Rannala, 2010; Yang & Rannala, 2014).

The gene flow interruption process is, however, not uniform across the genome. At intermediate speciation stages, loci involved in reproductive isolation (species barriers) experience little or no gene flow, whereas neutral loci can be freely exchanged between the diverging populations (Coyne & Orr, 2004, Figure 1b). The population genomic literature provides numerous examples of such mosaic diverging genomes. Genetic differentiation between malaria vectors *Anopheles gambiae* and *Anopheles coluzzi*, for instance, is strong in low‐recombining regions of the genome, but weak or inexistent at other loci due to recent gene flow (Miles et al., 2017). In this group, the true species tree is only supported by a minority of genes (Fontaine et al., 2015). The discovery of pervasive genome mosaicism—i.e., admixture between closely related species, or cryptic genetic background subdivisions within species—has raised complex issues regarding gene flow assessment (Sousa & Hey, 2013) and interpretation of so‐called Fst outliers or speciation islands (e.g., Bierne, Roze, & Welch, 2013, Cruickshank & Hahn, 2014, Ravinet et al., 2017, Wolf & Ellegren, 2017, ). A recent comparative study across 61 pairs of populations/species of animals (Roux et al., 2016) suggested that the rate of accumulation of species barriers, and therefore of gene flow interruption, is reasonably well predicted by the net amount of DNA sequence differentiation, that is, divergence minus polymorphism, often denoted *d*_A_. Gene flow was found to be high and fairly homogeneous across loci when *d*_A_ was below 0.5%, absent or highly reduced when *d*_A_ was above 2%, and typically heterogeneous across loci for intermediate *d*_A_ values.

Could the species problem therefore simply be solved by deciding on a threshold on *d*_A_, or on the portion of the genome experiencing gene flow? Probably not, unfortunately. First, not every scientist agrees that reproductive isolation is the main criterion to be used—hence the many species “concepts” (De Queiroz, 2007; Hey, 2006). Even if we recognize that gene flow is key, it is unclear whether the analysis of introgression patterns could provide a universal species delineation algorithm. One reason is that not only species barriers—in the sense of counter selection against hybrid/recombined genotypes—can affect introgression patterns between populations/species. The uneven distribution of segments of Neanderthal origin in the *sapiens* genome, for instance, is thought to primarily reflect a difference in the load of recessive deleterious mutations between the two populations, not epistatic interactions among loci (Harris & Nielsen, 2016; Juric, Aeschbacher, & Coop, 2016). A second reason is that introgression patterns partly depend on contingent aspects, such as the opportunity for two gene pools diverging in allopatry to experience secondary contacts. Geography matters and differentiated gene pools that occur in sympatry could be more likely to correspond to true species than allopatric ones. A third reason is that natural systems are complex and rarely fit the basic speciation genetic model of two genetically homogeneous entities that gradually diverge (Figure 1). There can be more than two gene pools that diverge/interact (e.g., Gladieux et al., 2015, Leroy et al., 2017), and not the same number/sets of loci might act as barriers between distinct pairs of gene pools.

All species barrier loci, finally, are not qualitatively equivalent. Some, but not all, are linked to conspicuous morphological traits, ecological differentiation and/or prezygotic isolation (Mérot, Salazar, Merrill, Jiggins, & Joron, 2017). Some, but not all, are geographically structured, across clines or among islands. Some, finally, are strong and irreversible, and will in the long run lead to complete reproductive isolation, while others are transient (Mallet, 2008). If species are defined as sets of co‐adapted alleles and traits in linkage disequilibrium, then it could be argued that what matters is how long such co‐adapted sets will escape extinction, or how strongly linked they are, rather than how much gene flow is currently ongoing at neutral loci (Mallet, 2008; Mallet, Besansky, & Hahn, 2016). Most specialists would presumably oppose the suggestion of lumping *Heliconius melpomene* and *Heliconius cydno* butterflies into a single species, even though 42% of their genome has been affected by recent gene flow (Martin et al., 2013). This logic, however, can reach its limits if pushed too far. Should we, for instance, split the morphologically distinct European crows *Corvus corone corone* versus *C. corone corvis* into two distinct species? Basically, a single two‐megabase genomic window—<1% of the genome—is resistant to gene flow between these two entities. This region contains genes for plumage colour and visual perception, which are likely involved in prezygotic isolation (Poelstra et al. 2014). So on the one hand, the situation in crows appears to be qualitatively similar to that in *Heliconius* butterflies, but on the other, it could also be described as just one assortative mating locus.

An additional element of complexity is that species barrier loci are not so easy to empirically identify, count and characterize, even with genome‐wide data sets. Barriers to gene flow are typically detected as genomic regions showing higher than average population differentiation—for example, Fst. However, not only differential introgression can generate across loci heterogeneity in population differentiation. Linked directional selection, in particular, is a major confounder of Fst scan approaches (Charlesworth, 1998; Cruickshank & Hahn, 2014). Both background selection and selective sweeps are expected to lower polymorphism and increase Fst in regions of low recombination and high gene density. Patterns of heterogeneous genome differentiation therefore reflect a combination of heterogeneous gene flow due to barrier loci and linked directional, the former, but not the latter, being relevant to speciation. The two effects are not easy to disentangle, which further complicates the species delineation problem from a practical point of view.

To summarize, even though we clearly have learnt much about the speciation process over the last decade thanks to genomic data, it seems that this knowledge has emphasized, rather than overcomes, the complexity of the species problem—a suggestion perhaps best illustrated by the Twitter identifier of one of the most eminent thinkers in the field (https://twitter.com/wtf_r_species).

## 4 WHY WE SHOULD DELINEATE SPECIES

So specialists seem to agree that delineating species can only be arbitrary, as both the distribution of variation in nature and the gene flow interruption process are multidimensional and continuous. Having to summarize this complexity as a mere list of species can appear pointless, or frustrating, to researchers interested in speciation. Non‐specialist scientists and the general public, however, need species as a simplified representation of natural variation. We have to name discrete biological entities, and taxonomy has to have a lowest‐level rank. A number of research fields, such as macroecology and macroevolution, rely on our ability to count species (Faurby, Eiserhardt, & Svenning, 2016; Isaac, Mallet, & Mace, 2004). That there are more species in tropical than temperate ecosystems, and more species of rodents than of primates, are essentially undisputed statements that likely reflect biological truths. The fact that it is hard to come up with a ubiquitous species delineation procedure does not render species a meaningless concept (Hey, Waples, Arnold, Butlin, & Harrison, 2003).

Inappropriate species delineation can have negative impacts on fundamental research. For instance, only recently was it discovered that the model ascidian *Ciona intestinalis* actually consists of two species diverged by ~15% at the genome level, now called *C. intestinalis* and *Ciona robusta* (formerly type A and B Brunetti et al., 2015, Nydam & Harrison, 2010, Roux, Tsagkogeorga, Bierne, & Galtier, 2013), whereas the morphologically distinct *Ciona roulei* is very close to, and probably a subspecies of, *C. intestinalis* (Malfant, Darras, & Viard, 2018). The substantial cell/developmental biology literature published on this taxon is actually a patchwork of studies on *robusta* (e.g., Oda‐Ishii et al., 2016), on *intestinalis *(e.g., Ryan, Lu, & Meinertzhagen, 2016), and on mixed samples (e.g., Esposito et al., 2017), so it is hard to differentiate experimental noise and biological variation.

Importantly, species boundaries also influence a number of applied issues, particularly wildlife conservation (Hey et al., 2003). Creating lists of endangered species is a major activity of conservation authorities. A number of agencies recognize management units at the population/subspecies level, and in principle conservation could be decoupled from taxonomy (Haig et al., 2006). In practice, however, taxonomy influences conservation. Managers at some point have to assign organisms to named entities, and in many cases, it is likely that different decisions will be taken depending on whether such entities are true species or just populations or ecotypes. Whether gene flow should be prevented (to avoid genetic pollution) or facilitated (to achieve genetic rescue), for instance, is a matter of debate and very likely influenced by the existing taxonomy (Allendorf, Hohenlohe, & Luikart, 2010; Frankham et al., 2012), with intraspecific gene flow often being considered as beneficial, and between‐species gene flow as a threat. Species delineation also has an obvious impact on species “evolutionary distinctiveness,” a statistics aiming at measuring the extent to which a given species is isolated in a phylogenetic tree, which has been proposed as a criterion for deciding on conservation priorities (e.g., Redding and Mooers 2006)—if an isolated species is split in two, each of the newly created species now has a close relative (Pavoine et al. 2005). Our assessment of the species richness of specific areas/ecosystems—which clearly depends on taxonomy—is also a key element in conservation policy.

Species boundaries, therefore, are arbitrary while having deep scientific and societal impacts, which is not a desirable situation. In the absence of an objective solution to the species problem, different scientists analysing the same data might come to distinct conclusions and consequently issue distinct recommendations. A related issue is that, because scientists do not use a unique species definition and delineation procedure, species boundaries in different taxa have different meanings. Taxonomy is heterogeneous.

The issue is particularly sensitive in endangered taxa, where species delineation can have immediate consequences on management decisions. Scientists are typically concerned by the preservation of the organisms they work on, and there might be a temptation to adjust species boundaries according to conservation priorities. The trend here could be towards over‐splitting of charismatic endangered organisms (Isaac et al., 2004). Taxonomic inflation is reinforced, in the genomic era, by the increasing availability of high numbers of genetic markers at affordable cost. As mentioned above, even moderate differences in allele frequencies between clusters of individuals will reach high statistical significance if genome‐wide data are analysed, further prompting taxonomists to rank such clusters at subspecies or species levels (Hey, 2009). Sequencing initiatives are unequally distributed among taxa, which is another reason for the imbalanced taxonomy.

I am here arguing that the existing across‐taxa heterogeneity in species delineation procedures is problematic, both for basic and applied research. There is no particular reason for prioritizing (or deprioritizing) oversplit taxa in the allocation of conservation resources. Furthermore, due to the lack of a ubiquitous species delineation procedure, if it seems that researchers consciously or unconsciously tend to propose a taxonomy that best matches their view on conservation, this could result in a long‐term discredit of the scientific discourse, while penalizing research fields that rely on taxonomy. The species problem, however complex, belongs to science. We need a taxonomy that best reflects the available information, and is influenced as little as possible by scientists' inclinations. We need a norm.

## 5 PROPOSAL: REFERENCE‐BASED TAXONOMY

Adopting a standardized procedure for species delineation unfortunately seems impossible, for the reasons considered above and elsewhere. How can a standard be defined when: (a) scientists disagree on the species definition, (b) speciation in nature can follow so many different routes, and (c) the size and nature of available data vary considerably in time and among taxa? As discussed above, a universal species delineation criterion probably does not exist. Still, recent studies have demonstrated the pertinence of comparisons between distantly related taxa as far as the species problem is concerned (Hey & Pinho, 2012; Riesch et al., 2017; Roux et al., 2016).

One suggestion would be to seek to progress towards taxonomic standardization via the use of reference systems. One idea would be to identify taxa in which large amounts of data are available, and species boundaries are consensual, or can be agreed on. Species delineation in any other taxon could thus be achieved so as to maximize consistency with the reference. Whatever criteria are deemed relevant to species delineation in one particular group should be assessed in the reference taxa, from which thresholds would be set based on the accepted taxonomy. One strong feature of this approach is its versatility. It is adaptable to all sorts and sizes of data, provided data of similar sorts and sizes are available in the reference system. This would also provide some objectivity and reproducibility in taxonomic decisions. One could write: “Taxon X was split into X1 and X2 because X1 and X2 are more differentiated than R1 versus R2 according to criterion C,” where R1 and R2 are two distinct reference species.

Although such a system would clearly not resolve everything, taking a comparative approach to the species delineation problem should help to converge towards a more standardized taxonomy, while avoiding incongruities (Loire & Galtier, 2017). It should be mentioned that taxonomy is already largely comparative, especially as far as higher order taxa are concerned. When deciding on whether a newly identified group of species deserves a genus or family level, taxonomists typically refer to well‐established genera and families in related taxa. This is probably true of the species rank too (e.g., see discussion in Nater et al., 2017), but the picture is more complex because of the diversity of prevailing approaches and disagreements on the species concept. For instance, recent suggestions of splitting *Giraffa camelopardalis* (giraffe, Fennessy et al., 2016) and *Chelonoidis nigra* (giant Galapagos tortoises, Poulakakis et al., 2015) into four and 13 distinct species, respectively, were based on very different arguments. These articles and the debates that followed (Bercovitch et al., 2017; Fennessy et al., 2017; Loire & Galtier, 2017) have very little in common. They illustrate how current taxonomy is influenced both by newly generated datasets and by scientists' views on what species are.

So which taxa should we use as a reference? “Many” is probably the best answer. An obvious candidate, to start with, would be our own species. A very large amount of morphological, ecological and genetical data is available in *Homo sapiens* and its close relatives. Regardless of the data set that is gathered in one’s favourite taxon, a comparable data set probably exists in humans and apes. Importantly, species boundaries among extant lineages in this group are consensual. Scientists agree that all human beings belong to the same species, even though significant morphological and genetic differences between human populations are documented. Scientists also agree that chimpanzees, bonobos, gorillas and other primates belong to species that differ from *H. sapiens*. So to illustrate my point, one proposal could be that, at least in vertebrates:

- Rule 1: Any set of entities showing more divergence/differentiation than observed between human and chimpanzee should be considered distinct species;
- Rule 2: Any set of entities showing less divergence/differentiation than observed between distinct human populations should be considered a single species.

How divergence/differentiation should be measured is clearly an important question, which relates to the species concept, and will probably keep finding new answers as science progresses. But whichever criterion one picks, ensuring that is applied the same way across taxa sounds like a minimal requirement.

Clearly, a large number of species delineation issues—the most difficult ones—cannot be solved by the two rules outlined above. This happens when the analysed entities are more differentiated than human populations, but less than humans versus chimpanzees, that is, the grey zone of speciation. So reference taxa at intermediate divergence levels would appear necessary. To follow‐up with the human example, a great deal of morphological and molecular data is appropriately available in *Homo neanderthalensis*, an archaic lineage of humans which offers a convenient point of comparison. There is some debate about whether Neanderthals formed a species different from *H. sapiens*, as first suggested based on morphology, or a distinct population from the same species. I suggest that we decide on this issue first and then propagate to other taxa via rules analogue to Rule 1 and Rule 2 above. This is an important decision also from an ethical standpoint, and not only scientists might have a word to say here. Based on the recent evidence that genes have more or less freely flowed between *sapiens* and *neanderthalensis* during their short period of contact (Harris & Nielsen, 2016; Juric et al., 2016), this author's opinion would be to lump them in a single species—and adapt Rule 1 and Rule 2 above accordingly.

The human/apes situation could be a useful reference when considering species delineation in other taxa of mammals and vertebrates. It is, however, unlikely to be pertinent across the whole tree of life. Morphological or geographical criteria that are relevant in primates, for instance, cannot be easily propagated to, for example, protists or bacteria. Additional references are to be identified in various phyla of plants, animals and microbes. Ideally, creating a public database dedicated to speciation and speciation genomics, with standardized fields (e.g., sample size, geography, data type, main summary statistics, PCA plots...), to which scientists could refer when interpreting their own data, would appear as a promising tool in order to progress towards a standardized taxonomy.

## CONFLICT OF INTEREST

None declared.
